# Coronary Flow Velocity Reserve Declines After Anthracycline Therapy in Breast Cancer Patients

**DOI:** 10.1016/j.cjco.2024.01.009

**Published:** 2024-02-03

**Authors:** Christopher Yu, Prajith Jeyaprakash, Koya Ozawa, Tomoko Negishi, Dhanusha Sabanathan, John Park, Jennifer Man, Anuradha Vasista, Faraz Pathan, Kazuaki Negishi

**Affiliations:** a Sydney Medical School, Charles Perkins Centre Nepean, Faculty of Medicine and Health, The University of Sydney, Sydney, New South Wales, Australia; b Cardiology Department, Nepean Hospital, Sydney, New South Wales, Australia; c Medical Oncology Department, Nepean Hospital, Sydney, New South Wales, Australia

## Abstract

Anthracycline therapy (ANT) is associated with cancer therapy-related cardiac dysfunction. Coronary flow velocity reserve (CFVR) has shown prognostic utility in non-cancer cohorts, but no data have been obtained in a cardio-oncology setting. We investigated the acute effect of ANT on CFVR in breast cancer patients. A total of 12 female breast cancer patients undergoing ANT had pre- and post-ANT CFVR assessment. A significant decline in CFVR occurred (baseline: 2.66 ± 0.41 vs post-ANT: 2.47 ± 0.37, *P* = 0.016). This prospective study is the first to identify ANT-related coronary physiology changes in humans. Further studies are required to determine their clinical significance.

## Graphical abstract

**Figure:**
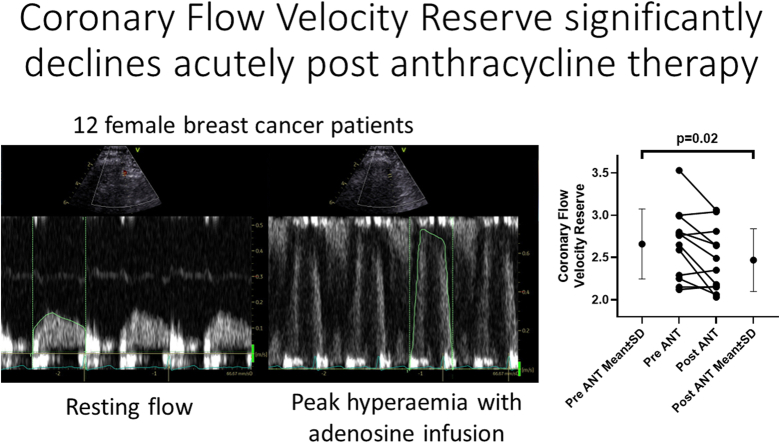

## Résumé

Le traitement par l’anthracycline est associé à une dysfonction cardiaque liée au traitement anticancéreux. La réserve de débit coronaire a démontré son utilité pronostique dans les cohortes sans cancer, mais aucune donnée n’a été obtenue dans un contexte de cardio-oncologie. Nous avons étudié l’effet aigu de l’anthracycline sur la réserve de débit coronaire chez des patientes atteintes d’un cancer du sein. La réserve de débit coronaire a été évaluée avant et après le traitement par l’anthracycline chez un total de 12 femmes atteintes d’un cancer du sein. Un déclin important de la réserve de débit coronaire est survenu (valeur initiale de 2,66 ± 0,41 par rapport à 2,47 ± 0,37 après le traitement par l’anthracycline, *p* = 0,016). Cette étude prospective est la première à déceler des changements dans la physiologie coronarienne liés à l’anthracycline chez les humains. D’autres études sont nécessaires pour en déterminer la portée clinique.

Anthracycline therapy (ANT) is associated with cancer therapy-related cardiac dysfunction (CTRCD). In swine models, ANT can impact invasively measured coronary flow reserve (CFR), but evidence in humans is limited.^1^ Coronary physiology can be measured noninvasively using coronary flow velocity reserve (CFVR) by transthoracic echocardiography.^2^ CFVR can be measured accurately with high reproducibility and can reflect invasive CFR.^2^ A recent meta-analysis demonstrated that reduced coronary flow is associated strongly with increased risk of all-cause mortality and major adverse cardiovascular events, demonstrating its role as a prognostic tool.^3^ CFVR has shown prognostic utility in an ischemic setting, but no data have been obtained in the cardio-oncology setting.^4^ We investigated the acute effect of ANT in breast cancer patients on CFVR.

## Methods

We obtained institutional research ethics board approval (reference number: 2021/ETH00406), and patients signed informed consent forms. Consecutive breast cancer patients referred for baseline echocardiography prior to ANT were prospectively enrolled in the study. Patients with a history of known coronary heart disease, lung disease, or contraindication to adenosine were excluded. The baseline echocardiogram was used as a control and was compared to post-ANT echocardiograms, which were planned to occur within 1 week of ANT completion. All the images were acquired with the highest possible frame rate and were measured offline by a reader who was blinded to the patient information and time point. Coronary flow was imaged from the mid-distal portion of the left anterior descending (LAD) artery in a modified low parasternal long-axis view, under the guidance of colour Doppler flow mapping. At each time point, 3 optimal profiles of peak diastolic Doppler flow velocities were acquired, and the average of 3 measurements was used. Hyperemia was achieved by an adenosine infusion (140 microgram/kg/min). The CFVR was calculated as the hyperemic/rest ratio of diastolic LAD peak flow velocity (PFV) pulsed-Doppler assessment of LAD flow. A 3-dimensional full-volume acquisition of the left ventricle with a matrix array transducer with the highest possible volume rate was attempted in all patients. If 3-dimensional left ventricular (LV) volumes were unable to be acquired, 2-dimensional LV ejection fraction (EF) was measured by the biplane method of discs, using apical 4- and 2-chamber images in keeping with guidelines.^5^ LV global longitudinal strain (LVGLS) analyses were performed by using a semiautomated speckle tracking technique (EchoPAC, GE Healthcare, Milwaukee, WI).

Continuous variables were compared using the paired *t* test or the Friedman test, as appropriate. All cases were chosen to assess interobserver variability, which was assessed using an intraclass correlation coefficient using a 2-way mixed model with absolute agreement between measures. Statistical significance was defined as *P* < 0.05.

## Results

**Figure 1:**
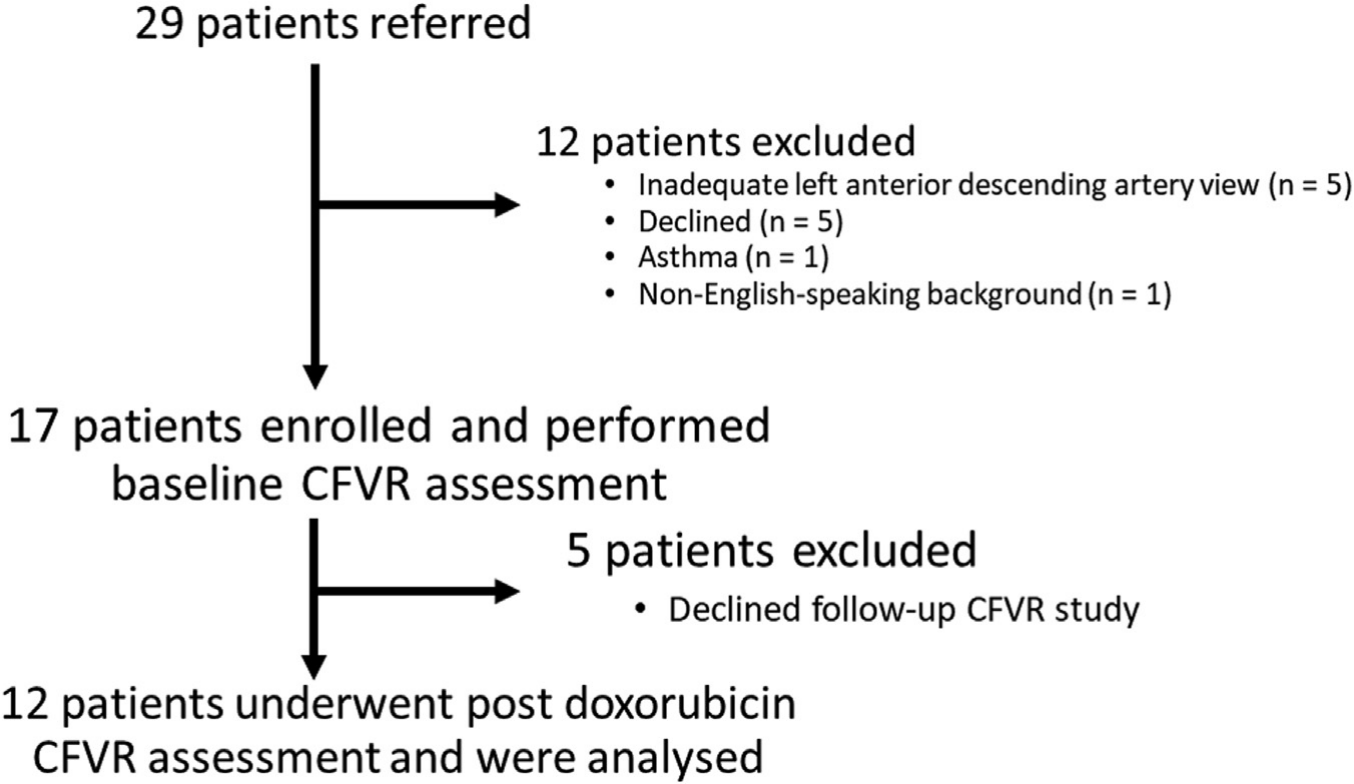
Study flowchart. CFVR, coronary flow velocity reserve.

**Figure 2:**
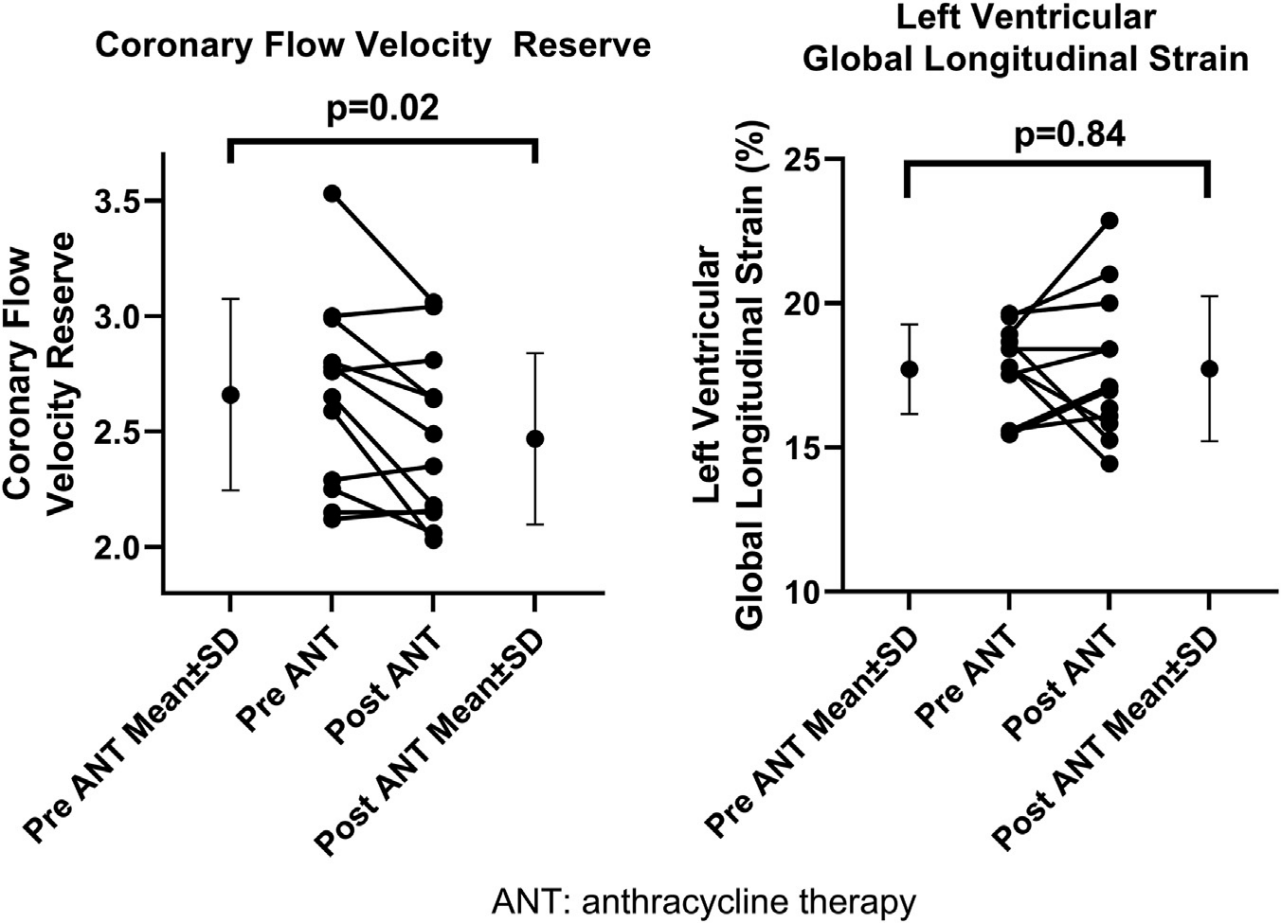
Changes in coronary flow velocity reserve (CFVR) and left ventricular global longitudinal strain pre– and post–anthracycline therapy. ANT, anthracycline therapy; SD, standard deviation.

Of the 29 eligible patients referred, 12 had baseline and post-ANT CFVR measurements performed, with 17 excluded (n = 5 declined participation; n = 5 declined post-ANT CFVR study; and n = 5 had an inadequate LAD view; Fig. 1). All the participants were female patients, with a mean age 56 ± 14 years, and received a cumulative doxorubicin dose of 240 ± 0 mg/m^2^ (4 cycles of 60 mg/m^2^). Other demographic information can be found in Table 1. All patients had normal baseline LVEF (59% ± 5%), and an LVGLS of (-17.7% ± 1.6%), with normal LV diastolic function (Table 1**)**. No changes occurred in vital signs, LVEF (59% ± 5% vs 59% ± 5%, *P* = 0.82), LVGLS (-17.7% ± 1.6% vs -17.9%, *P* = 0.84; Table 1), or diastolic parameters post ANT. However, postANT, CFVR significantly declined compared to baseline (baseline: 2.66 ± 0.41 vs post-ANT: 2.47 ± 0.37, *P* = 0.016; Fig. 2). No significant change occurred in the peak stress LAD PFV (baseline: 78 ± 23 vs post-ANT: 82 ± 25 cm/s, *P* = 0.41), and a trending but nonsignificant increase occurred in resting LAD PFV (baseline: 29 ± 5 vs post-ANT: 33 ± 7 cm/s, *P* = 0.07). The intraclass correlation coefficients are for interobserver variability for baseline resting LAD PFV, baseline peak stress LAD PFV, post-ANT resting LAD PFV, and post-ANT peak stress LAD PFV, respectively, were 0.97 (0.91, 0.99), 0.99 (0.99, 1.00), 0.99 (0.97, 1.00) and 0.99 (0.98, 1.00), demonstrating excellent interobserver class reproducibility.

**Table 1:** Baseline demographics and pre– and post–anthracycline therapy measurements in vital signs and echocardiographic parameters

Patient characteristics and parameters	Baseline measurement	Post–anthracycline therapy measurement	*P*
Female	12 (100)	—	—
Age, y	56 ± 14	—	—
Cumulative doxorubicin dose, mg/m^2^	240 ± 0	—	—
Height, m	1.64 ± 0.10	—	—
Weight, kg	82 ± 24	—	—
Body mass index, m/kg^2^	30.2 ± 7.8	—	—
Smoking history	5 (42)	—	—
Hypertension	4 (25)	—	—
Diabetes	1 (8)	—	—
Hyperlipidemia	1 (8)	—	—
Left-sided breast cancer	5 (42)	—	—
Systolic blood pressure, mm Hg	128 ± 19	131 ± 18	0.59
Diastolic blood pressure, mm Hg	84 ± 9	82 ± 14	0.62
Heart rate, bpm	72 ± 10	81 ± 14	0.12
Left ventricular ejection fraction, %	59 ± 5	59 ± 5	0.82
Left ventricular global longitudinal strain, %	17.7 ± 1.6	17.9 ± 2.5	0.84
E velocity, cm/s	60 ± 9	61 ± 14	0.67
A velocity, cm/s	66 ± 18	69 ± 14	0.53
E/A ratio	0.97 ± 0.32	0.91 ± 0.31	0.63
Left atrial volume indexed, mL/m^2^	27 ± 6	26 ± 5	0.53
Average E/e’	7 ± 1	8 ± 2	0.10
Resting LAD peak flow velocity, cm/s	29 ± 5	33 ± 7	0.07
Peak stress LAD peak flow velocity, cm/s	78 ± 23	82 ± 25	0.41
Coronary flow velocity reserve	2.66 ± 0.41	2.47 ± 0.37	0.016

Values are n (%) or mean ± standard deviation, unless otherwise indicated.

LAD, left anterior descending.

## Discussion

This prospective study is the first in humans to investigate coronary physiology changes in patients with breast cancer treated with ANT. We demonstrated that CFVR significantly declines acutely post completion of ANT in these patients. Additionally, the decline in CFVR occurred before any decline in LV systolic or diastolic parameters. The decline in CFVR may partly explain the mechanism of ANT-related CTRCD and a possible method for earlier CTRCD detection. Galán-Arriola et al. demonstrated that CFR declined early after a course of doxorubicin in pigs, as it did in our study.^1^ However, our decline was modest, at 7%, vs 34%. This difference could be due to numerous factors, such as the intracoronary injection of ANT with increased dosing and duration, as well as the invasive assessment of CFR in pigs. The decline in CFVR in our study appears to be driven by the increase in resting LAD PFV. Swine histopathology demonstrated that the coronary arterioles are damaged, and the capillaries are preserved after doxorubicin treatment.^1^ This evidence suggests that ANT leads to mild alterations in microvascular function, potentially due to edematous changes in the myocytes and endothelial damage in the arterioles (40∼100 μm). This situation necessitates the dilation of capillaries (< 40 μm), even at rest, to maintain the pressure gradient between the epicardial arteries and capillaries, thereby increasing resting coronary flow of the epicardial artery. However, these changes, being early and mild, do not impair the capillaries’ ability to achieve maximal dilation in response to adenosine administration. Thus, the peak stress LAD PFV did not change significantly. Of note, no significant change in heart rate was observed (*P* = 0.12).

Our results are not conclusive, rather hypothesis generating, which warrants further investigation. The curent literature has shown that CFVR changes can occur prior to other echocardiographic parameters such as LVEF.^6^ Additionally, for every 0.1 unit decline in CFR, a 16% increase occurs in hazard of death.^3^ Unfortunately, our pilot study was not designed to identify these changes; however, the decline in CFVR supports further investigation into this area of cardio-oncology.

Lastly, this study has demonstrated that CFVR can be measured in a female breast cancer cohort, with excellent reproducibility. This finding allays concerns regarding echocardiographic measured CFVR in this patient population, as echocardiographic imaging can be complicated due to breast-related surgery.

## Limitations

First, the small sample size restricted the power to examine certain factors that impact CFVR. Although differences occurred in some parameters, these are of unclear clinical significance. Larger studies are required with different ANT regimens to determine if these results are applicable in other cancer groups. Second, the short follow-up period means that only the short-term effects of doxorubicin were analyzed. Further studies are needed to determine the long-term impact of anthracyclines on the coronary microcirculation, and whether the early changes in CFVR occur prior to other echocardiographic parameters. Lastly, we can describe changes in only CFVR; no other parameters of coronary physiology, such as the index of microcirculatory resistance, were assessed. Nonetheless, CFVR alone is a robust prognostic tool.

## Conclusion

Among patients with breast cancer, CFVR was significantly reduced acutely after the completion of ANT. Further studies are required to determine the exact mechanisms for this change, its clinical significance, and whether it is replicated in other cancer cohorts treated with anthracyclines.
